# Staff experiences of closing out a clinical trial involving withdrawal of treatment: qualitative study

**DOI:** 10.1186/s13063-017-1813-y

**Published:** 2017-02-07

**Authors:** Julia Lawton, David White, David Rankin, Jackie Elliott, Carolin Taylor, Cindy Cooper, Simon Heller, Nina Hallowell

**Affiliations:** 1 Centre for Population Health Sciences, University of Edinburgh, Edinburgh, UK; 2 Clinical Trials Research Unit, University of Sheffield, Sheffield, UK; 3 The Sheffield Diabetes and Endocrine Centre, Northern General Hospital, Sheffield, UK; 4 Academic Unit of Diabetes, Endocrinology and Metabolism, University of Sheffield, Sheffield, UK; 5 Ethox Centre, Nuffield Department of Population Health, University of Oxford, Oxford, UK

**Keywords:** Clinical trials, Closeout, Withdrawal of treatment, Staff experiences, Ethical issues, Emotion, Qualitative

## Abstract

**Background:**

The ending of a clinical trial may be challenging, particularly if staff are required to withdraw the investigated treatment(s); however, this aspect of trial work is surprisingly under-researched. To address this gap, we explored the experiences of staff involved in closing out a trial that entailed withdrawal of treatment (insulin pumps) from some patients.

**Methods:**

Interviews were conducted with *n* = 22 staff, recruited from seven trial sites. Data were analysed thematically.

**Results:**

Staff described a myriad of ethical and emotional challenges at closeout, many of which had been unforeseen when the trial began. A key challenge for staff was that, while patients gave their agreement to participate on the understanding that pump treatment could be withdrawn, they often found themselves benefitting from this regimen in ways they could not have foreseen. Hence, as the trial progressed, patients became increasingly anxious about withdrawal of treatment. This situation forced staff to consider whether the consent patients had given at the outset remained valid; it also presented them with a dilemma at closeout because many of those who had wanted to remain on a pump did not meet the clinical criteria required for post-trial funding. When deciding whether to withdraw treatment, staff not only had to take funding pressures and patient distress into account, but they also found themselves caught between an ethic of Hippocratic individualism and one of utilitarianism. These conflicting pressures and ethical considerations resulted in staff decision-making varying across the sites, an issue that some described as a further source of ethical unease. Staff concluded that, had there been more advanced planning and discussion, and greater accountability to an ethics committee, some of the challenges they had confronted at closeout could have been lessened or even prevented.

**Conclusions:**

The same kinds of ethical issues that may vex staff at the beginning of a trial (e.g. patients having unrealistic expectations of trial participation; staff experiencing conflicts between research and clinical roles) may re-present themselves at the end. To safeguard the wellbeing of staff and patients, greater planning, coordination and ethical oversight should go into the closeout of trials involving withdrawal of treatment(s).

**Trial registration:**

International Standard Randomised Controlled Trials Number (ISRCTN) Registry, ISRCTN61215213. Registered on 11 May 2011

**Electronic supplementary material:**

The online version of this article (doi:10.1186/s13063-017-1813-y) contains supplementary material, which is available to authorized users.

## Background

Research undertaken with trial participants can be used to improve recruitment and consenting procedures, offer insights into how trial interventions are received, aid interpretation of trial findings and provide recommendations for conducting future trials [1]. To date, this work has mostly focused on patients’ understandings and experiences. However, the value of including staff perspectives has also been recognised, with studies highlighting how staff’s attitudes and understandings can impact on recruitment, delivery of trial interventions and, more latterly, trial outcomes [2–5]. Indeed, studies undertaken with trial staff have revealed a complex picture, wherein practical issues, such as having adequate resourcing to undertake trial work, are interwoven with individuals’ own values, clinical judgment and ethical considerations [6–8]. For instance, studies have shown that, while trial staff may adhere to notions of community equipoise, they may not be in individual equipoise [9], and this may result in them not approaching certain individuals if they are concerned that this might result in them being randomised to treatments that staff see as inappropriate in their particular case [5–7, 10]. Staff may also be reluctant to approach certain individuals because of concerns about upsetting or overburdening them [4, 6], reluctance to admit that they are uncertain about the effectiveness of treatments [6] and, relatedly, worries about compromising an on-going therapeutic relationship [4, 6]. Indeed, it has been shown that because (trial) research and clinical care have different epistemological underpinnings—the former being hypothesis driven, the latter needs driven—trial staff commonly experience conflicts between research and clinical roles [11–13]. These conflicts may not only impact on recruitment [5, 7, 14], but also staff members’ adherence to trial protocols as these require them to follow standardised procedures rather than provide patients with individualised, tailored care [2, 8]. As research further suggests, staff's recruitment practices and adherence to trial protocols may also be influenced by individual factors, such as their level of clinical experience [2] and contextual factors, such as the size and organisation of their clinics [8, 15]. Indeed, it is because trial recruitment and delivery practices can be influenced by individual and contextual factors that commentators have questioned whether trial interventions will work in the same ways and have the same clinical impact when rolled out into routine clinical practice [1, 15].

Alongside practical and ethical issues, a small body of work has drawn attention to attendant emotional challenges arising for trial staff. For instance, studies exploring the perspectives of recruiting staff have shown that individuals may struggle emotionally as well as intellectually with concepts such as equipoise [7]. Staff may also find recruitment anxiety provoking because of experiencing conflicts between research and clinical roles [6], worrying about upsetting potential recruits [4] or not meeting recruitment targets [16]. While the main focus of this work has been on recruitment, it has also been shown that emotional challenges can extend into subsequent stages of a trial, such as when staff have to notify patients about the outcome of randomisation [16] or encourage patients to remain in a trial when staff do not see this as being in their best clinical interests [10].

To summarise, research has highlighted the practical, ethical and emotional challenges that may be encountered by trial staff. In doing so, this research has also underscored the importance of staff being given appropriate training, education and emotional support to undertake recruitment and deliver trial interventions in consistent and unbiased ways [4–7, 9, 16]. However, one key aspect of trial work remains absent from this research: the closeout or ending of a trial. This is a striking omission given that, as some commentators have noted, the ending of a trial is likely to give rise to practical and ethical challenges, especially if this requires staff to withdraw treatment and care from patients [17]. Hence, this article seeks to address this gap by reporting findings from an interview study involving staff who were involved in closing out a trial that required them to withdraw the study treatment, insulin pumps, from some of those who took part.

### The REPOSE trial

The REPOSE (Relative Effectiveness of Pumps Over MDI and Structured Education) trial was a parallel group, cluster randomised controlled trial (RCT), conducted in eight centres in the UK between November 2011 and June 2015 [18]. In the UK, where health care is free at the point of delivery, the majority of adults with type 1 diabetes use a multiple daily insulin injection (MDI) regimen and strict clinical criteria, as outlined in the NICE (English) and SIGN (Scottish) guidelines, normally have to be met for patients to be eligible for an insulin pump. The two main criteria for pump eligibility are: (1) attempts to achieve target haemoglobin A1c (HbA1c) levels with MDIs result in the person experiencing disabling hypoglycaemia or (2) HbA1c levels have remained high (that is, at 8.5% or above) on MDI therapy despite a high level of care. As reported in the REPOSE trial protocol [18], a key reason for the restricted use of insulin pumps in the UK is because pumps are a much more expensive option than an MDI regimen. Also, at the time the trial was developed, there was insufficient evidence to approve extension of pump therapy to a wider population; indeed, this was a key rationale for the REPOSE trial being undertaken [18].

The REPOSE trial was designed to determine whether pump therapy provides added benefit compared to optimised MDI therapy after attending a structured education course [18]. The pumps were provided free of charge by a pharmaceutical company with a 2-year warranty and the pump consumables were funded at a local level [e.g. by Clinical Commissioning Groups (CCGs), which are clinically led statutory National Health Service (NHS) bodies responsible for the planning and commissioning of health care services for their local area]. Again this funding was restricted to a 2-year period, as the CCGs (or NHS boards in Scotland) did not want to commit to providing funding beyond the duration of the trial. Hence, if clinical staff decided that a patient should remain on an insulin pump following closeout from the trial, they had to make a case for local (e.g. CCG) funding to do this, in much the same ways as they are required to do for patients they wished to move onto pump therapy in routine clinical practice.

To be eligible for the trial, patients could not have used a pump in the previous 3 years or had a preference for pump therapy over a MDI regimen. Patients were ineligible if the local investigator determined that they had a strong clinical need for pump therapy, in line with NICE or SIGN guidelines (e.g. due to recurrent disabling hypoglycaemia). Checklists were used to ensure patients met all trial eligibility criteria, and those taking consent (local investigators or diabetes educators) discussed key aspects of the trial to help ensure that patients understood that, because of the restricted nature of the funding, individuals randomised to pump therapy could have this treatment withdrawn at the end of the trial.

Following randomisation, recruits attended a 5-day structured education course called DAFNE (Dose Adjustment for Normal Eating) to receive comprehensive instruction on how to adjust insulin doses to achieve optimal blood glucose control. These courses were delivered by diabetes educators (a diabetes specialist nurse and a dietician), one or more of whom also took responsibility for recruitment in each site, supported by the local investigator (a diabetes consultant) and a central Clinical Trials Unit (CTU), which was responsible for the trial’s overall management. Participants remained in the trial for 2 years and received their diabetes clinical care from the secondary care centres they usually attended (in practice, this often meant receiving care from the same staff who were responsible for delivering the trial). Participants attended trial appointments, delivered by the educators, at 6, 12 and 24 months, in order for clinical and psychological data to be collected.

Prior to closeout, each REPOSE site was advised, as per the trial’s Standard Operating Procedure (SOP), to make site-level decisions about which patients should remain on a pump or be returned to an MDI regimen. Staff were also reminded that they would have to find local funding for any individuals they wanted to keep on pump therapy. The SOP also mandated that patients could not be told whether they would remain on pump therapy until after final trial data had been collected, to avoid biasing trial data collection; specifically, patients’ responses to self-completion questionnaires that assessed their treatment satisfaction and quality of life.

This interview study was developed after closeout had begun in some sites and anecdotal reports had been received from some staff that they were encountering difficulties withdrawing pump therapy from some patients. A key aim of this study was to understand and explore staff experiences of closing out the REPOSE trial to provide recommendations for training and/or support that could be offered to staff involved in the closeout of future trials requiring withdrawal of treatment(s). Ethical approval for the study was granted by the Centre for Population Health Sciences Ethics Review Group, the University of Edinburgh, at the start of June 2015.

## Methods

Following receipt of ethical approval, recruitment was undertaken in seven of the eight REPOSE sites, which were based in England and Scotland; the eighth site was not included as it was added at the end of the trial and only hosted a small number of patients. All staff who had been involved in closeout (diabetes consultants and diabetes educators) were sent recruitment packs and invited to opt in; of the 24 staff approached, 23 opted in and 21 were interviewed (see Additional file 1: Table S1).

Interviews took place from the end of June 2015 to the end of August 2015, by which time the trial was in the final stages of closing out. Interviews were conducted by NH, an experienced qualitative researcher who was not a member of the trial team. Interviews were informed by a topic guide developed in light of literature reviews and initial reports from staff and revised in light of emerging findings. Key areas explored included: previous experiences of delivering clinical trials, experiences of recruiting and consenting into REPOSE, preparation and planning for closeout, how decisions about treatment allocation were made at the end of the trial and by whom, experiences of undertaking end-of-trial appointments and (if relevant) of withdrawing treatment, how staff felt the closeout of trials involving withdrawal of treatment(s) could be improved and how any of the ethical challenges they had encountered at closeout could be lessened or prevented in future trials. Interviews lasted ~60-90 min and were digitally recorded (with consent) and transcribed in full for in-depth analysis. By the time recruitment and interviewing were stopped, data saturation had been achieved, that is no new findings were identified in new data collected.

The interviews were analysed thematically by JL and NH using principles informed by the method of constant comparison [19]. JL and NH read all interviews through repeatedly before cross-comparing them to identify issues and themes that cut across different individuals’ accounts. These individuals then met to discuss their interpretations and reach agreement on key findings. Following this meeting, a decision was made to undertake an additional (comparative) analysis of staff interviews according to the trial site to which they belonged, as the initial analysis had made it apparent that staff practices and decision-making at closeout were often mediated and informed by site-specific (i.e. contextual) factors. JL and NH then developed a coding frame that captured cross-cutting themes together with the descriptive data needed to allow a ‘thick description’ [20] of the findings to be provided to readers that was sensitive to contextual factors. A qualitative analysis software package (NVivo10) was used to facilitate data coding and retrieval; to maximise rigor, both JL and NH were involved in the data coding process. Coded data sets were subjected to further analyses to enable more nuanced interpretations of the data to be developed and to select illustrative quotations and descriptive data used as part of the data reporting process.

To safeguard participants’ confidentiality, pseudonyms for individuals Dr (Diabetes Consultant) or Ed (Diabetes Educator) are used, with (A-G) indicating the site to which they belonged.

## Results

Staff provided very rich, candid and sometimes emotionally charged accounts of their experiences of closing out the REPOSE trial. As well as reinforcing earlier anecdotal reports that withdrawing pump therapy could be challenging, their interviews revealed big variations in staff practices and decision-making between (and sometimes within) the different trial sites. In some sites, staff kept most or all of their patients on pumps irrespective of whether they had gained clinical benefit from using this regimen, whereas in others pump therapy was withdrawn unless a clear clinical benefit, in line with NICE or SIGN criteria, could be evidenced. Not only did these variations in decision-making across (and sometimes within) the different sites come as a surprise to most staff, they also described them as having been a source of friction. Staff accounts also made apparent that, in making their decisions about whether or not to withdraw pump therapy, they had had to confront ethical and other challenges that they saw as having arisen directly from the trial, some of which had been unforeseen when the trial first began. Below, we explore these findings in more detail before considering learning points that staff were keen to share to improve the (ethical) conduct of future trials where treatment(s) might be withdrawn.

### Variations in practices and decision-making at the end of the trial

The variations in practice and decision-making described above, as some staff noted, only became apparent several months after closeout had begun when, in a monthly trial investigator meeting, a couple of staff talked about the difficulties they had encountered taking individual patients off pumps. Indeed, as Dr G2 noted, it was not until these difficulties had been raised that they had begun to question their assumption that co-investigator colleagues would “fiddle it for the patient” as they had done and allow those who wished to do so to remain on pump therapy following closeout from the trial:

“I was a bit taken aback when [colleague’s name] said somebody had walked out and I was also slightly taken aback when [another colleague’s name] said she’d stopped the pump and it hadn’t gone down too well, so clearly others were following the letter of the procedure, whereas I never thought I would, I thought I’d fiddle it for the patient because, I mean there are NICE guidelines, but you can get anybody on a pump if you really want…I mean you can call me a big softy, but I think we owe it to our patients to do the best by them. They live with diabetes 24 hours a day and it’s terribly demanding and it’s rotten for people and I think they should be given the support and if they want to keep the pump they should keep the pump. And it’s worth remembering that the technology in diabetes is pretty good value compared to some very expensive cancer treatments which may only buy a couple of months of extra life…So I think that I feel strongly that anything which makes life a bit better is probably worth it, and if we can get round the rules if it’s for the benefit of the patient, I’d always do that.”

This individual’s perspective stood in notable contrast to that of Dr F, who had removed pumps from the majority of patients they saw, and who, to justify their decision-making, highlighted a personal and ethical standpoint that was cognizant of what they saw as broader financial difficulties within the NHS and, hence, the need to allocate scarce resources in prudent ways:

“So I feel there are people who need a pump because without it they’re never going to get their diabetes right. And there are people who would like one because it might make it easier or it’s more convenient or for a variety of reasons. And my personal view would be that at the moment the NHS probably can’t afford to provide pumps for the latter.” (Dr F)

As Dr F’s comments served to highlight, contextual factors; specifically, availability of funding for pumps, could have a substantial impact on staff decision-making at the end of the trial. Indeed, as the analysis of the interviews made apparent, staff decision-making was broadly informed by whether they belonged to sites where they perceived there to be easy access to pump funding at the end of the trial (sites B, C, D, E) or those where access to NHS funding was much more limited (sites A, G, F). Dr G1 belonged to one of the latter sites, where, as this health professional described, local funding constraints, together with the limited availability of staff with pump training, had had a substantial impact on their decision-making. This, as Dr G1 described, had been to withdraw pump therapy unless a clear clinical benefit could be demonstrated that allowed a case to be made to the local CCG for on-going funding:

“If pumps were cheaper, if we had more staff who knew how to help patients, then there would be more pump use so… there were lots of kind of context to my decisions.” (Dr G1)

Indeed, this health professional went on to reflect on how, due to these contextual factors, they had had to “act as a filter” at the end of the trial because quality of life was not a criteria that they could use to apply for pump funding at their site:

“…the bottom line is that we can’t apply for funding based on quality of life. So no matter how much the patient tells us that they feel better, the clinician’s not interested in that at all. So whilst obviously I feel that quality of life is really important to patients and I would like to be able to support them in that, the rules don’t let me.” (Dr G1)

Similar “hard-nosed” (Ed A1) decisions were described by staff in site A where, again, due to the limited NHS funding available for insulin pumps in their region, staff said their decision-making had, by necessity, been “pretty cut and dried…unless there was a medical reason or unless they met NICE [criteria] already from a hypo[glycaemia] point of view, they had to come off.” (Ed A2)

In other sites, as already indicated, pump funding was more abundant at the time of closeout. In three cases this was due to the sites being based in Scotland and the Scottish government providing a substantial but temporary injection of funding for insulin pumps, which had happened to coincide with the ending of the trial: “so politics, you don’t look a gift horse in the mouth—we just said ‘yes, we’ll take these thank you very much” (Dr B). In the fourth site, as the lead investigator described, “we don’t really have an issue with funding for pump therapy. We never have had because historically, when primary care organization first came into existence, we already had probably 80 people on insulin pump therapy. So they just picked up the costs of that and subsequently they’ve just regarded insulin pump therapy as something they just fund really” (Dr C).

Because funding for insulin pumps was not so restricted in these sites at the time of closeout, staff, including Dr E, described how, when they were making decisions, “we knew there wasn’t going to be any immediate problem here about patients giving the pump back at the end of the study” (Dr E). As a consequence, staff also noted how it had been possible for them to base their decision-making on “much softer criteria” (Dr C), which took patients’ own preferences and quality of life considerations into account. Indeed, in such sites, staff, including Dr D, described how they had used diffuse and broad-ranging concepts of “doing well” to inform their decisions, which extended beyond the clinical eligibility criteria outlined in NICE/SIGN guidelines:

“It’s interesting isn’t it, so doing well might be having good blood glucose control. But doing well might just be engaging with their diabetes better than they did before. So, you know, we had a couple of quite chaotic people who don’t have perfect glycaemic control, but they’re testing [their blood glucose levels], they’re entering information [into the pump] and they’re keeping in touch with us in a way that before they weren’t. So I guess doing well can be something over and above what their blood sugar’s telling us.” (Dr D)

To justify and explain leniency in their decision-making at the end of the trial, some such staff also highlighted discrepancies between the care they had been able to give patients within the confines of the trial and that which they were able to provide in everyday clinical practice within their particular sites. Specifically, these staff members described how, when funding had permitted, they had tended to give patients who had not experienced clinical improvement “the benefit of the doubt” and “a second chance” by allowing them to remain on pump therapy, but with greater clinical support than had been permitted within the constraints of the trial protocol. This included Dr D who described how, in their site, they had “continued pumps in most who wanted to, even in the few we were slightly worried about” because “I am not sure our pump group in RESPOSE did as well as our [patients on] pumps do in our normal service because we didn’t put in the same amount of follow-up as we do for people who go onto pumps in our own service.” A similar course of action was reported by Dr C who described having allowed some patients who had actually experienced a deterioration in their blood glucose control to remain on pump therapy because they had wanted to give them “a proper chance, with very intensive management which, probably within the constraints of the study, I don’t think would have been appropriate” (Dr C).

### Staff reflections on variations in end-of-trial practices

The above variations in practice not only came as a surprise to most staff, the vast majority also described them as having been a source of discomfort or, more specifically, of ethical unease. In some cases, staff, including Dr C, simply expressed a general sense of discomfort that such disparities had existed, wherein: “it’s perhaps unfortunate that, you know, our pump users have virtually all carried on with the pump, whereas perhaps (other) pump users have all gone back onto MDI” (Dr C). In others, staff conveyed more candid views, by suggesting that colleagues at other sites might have taken an easy route out, and side-stepped some of the difficulties they had had to confront, by allowing most or all of their patients to keep their pumps:

“We had a telephone conference and I got the impression from that, that certainly some of the other centres hadn’t really thought about it. They just left the patients on their pumps. Now that’s my perception. But I felt, I felt really unsupported. The big—when I really got upset—this is turning into a psychotherapy session—when I really got upset was after the telephone conference, when people kind of said: ‘well we just didn’t do that’. And I think yeah—so that was difficult, because I felt we’d done something different. And we had followed the protocol…But it didn’t sound as though that had necessarily been the experience elsewhere.” (Dr F)

As well as questioning the practices of colleagues in other sites, some individuals indicated that tensions had developed within their own teams when it was felt that decision-making had been guided by inconsistent criteria or, as Ed B1 intimated, it became apparent that team members might have had different—and potentially conflicting—values:

“…he [Consultant’s name] certainly came back and told us all, you know, what his decision was, you know, he was happy for them all to stay on pumps…in the end it was his clinical decision but some of us had reservations. I mean the pump therapy’s it’s obviously an expensive you know, treatment to be using with patients…I just wonder with some of them is that the best use of resources?” (Ed B1)

Others still voiced underlying ethical concerns that, due to variations in how decisions were made, patients had not been given fair and equal access to (scarce) resources at the end of the trial:

“…to a certain extent still, pumps and funding for various things is a post-code lottery. As you know, different centres did do things in different ways. And so I think, I don’t know, potentially, you could kind of think, ‘well that’s not fair if one centre is just, had kept everyone on and we haven’t'...And I think the fact we did have to do it this way, you think, ‘oh that’s a bit annoying’. And you felt bad for the patients.” (Ed A2)

Indeed, alongside this concern, which was seen as having been symptomatic of a broader post-code lottery, staff identified a number of other issues at closeout that they saw as having arisen directly from the trial. As will now be considered, these presented a number of ethical and emotional challenges to staff, which, in the absence of advance planning and discussion across the trial sites, they had attempted to address in a variety of ways and with what they saw as varying degrees of success.

### Ethical and emotional challenges arising from the trial

As described earlier, to be eligible for REPOSE, patients could not have a treatment preference; patients also gave their consent on the understanding that pump therapy could be withdrawn at the end of the trial. However, staff who had had contact with patients during the trial noted that, as the trial progressed, most patients “became very fond of their pumps” as Ed E1 put it and, hence, as Ed B1 elaborated, they had also made it clear that they had wanted to keep them:

“They were absolutely fine at the start, they’re like, ‘yeah, yeah whatever’. They didn’t seem perturbed about that at all…that was to change as time went on. You know if they’d requested to see us, or at the kind of routine REPOSE follow-ups, when we asked how they were feeling about the pump, they all reported that they loved the pump, that they felt it was making their life so much easier and that they couldn’t imagine going back to having to inject multiple times a day. So they were all very vocal that they really wanted to stay on their pump, and that they would be prepared to fight for it if needs be.” (Ed B1)

As these staff went on to suggest, while some patients might have concealed a preference for a pump when they were recruited, others appeared to have gained benefits that they could not have necessarily foreseen. Such benefits, as Ed B1 observed, were principally those relating to improvements in their quality of life because pumps eased the burden of having to administer multiple daily injections. As some staff further reflected, this had presented a predicament and caused them anxiety at the end of the trial because, while patients had benefitted from using a pump, many had not done so in the clinical ways that could have been used to make a case to the local NHS funder for on-going use of this regimen.

“…because we did see patients who things have improved to, but the pumps, the background rates were very flat so you could do the same with injections. But they all liked the pump because there’s no injections…But it made it tricky at the end when we had to say to them: well actually you can achieve this with injections…The trial didn’t take into account the fact that they were happier on it. They felt calmer or you know, sort of all those quality of life things.” (Ed F4)

“I thought it [close-out] would be uncomfortable because the NICE criteria don’t talk about quality of life…I was thinking: well there’s going to be lots of patients that say they feel better. But actually have they hit any of the hard end points that help me say: yes you do need now to continue on pump.” (Dr G1)

Others reflected upon how experiences of observing patients become more worried and distressed about the possible removal of the pump as the trial progressed had forced them to question whether the consent they had given at the outset remained valid, and, hence, whether it was ethical to withdraw pump therapy despite patients having originally agreed to this. Indeed, some health professionals went as far to question whether patients had ever really been in a position to make a properly informed decision, given their lack of experience with pump therapy prior to the trial, a situation that led some to suggest that patient anger and upset would be justifiable in the event of a pump being withdrawn:

“So basically the patient had taken the decision to go on knowing that they were probably going to have to come off at the end of two years. So that kind of eased my conscience a bit in that the patients had made that decision themselves. But again, cause I had that longer experience in pumps and I knew what I’d seen, I just knew how awful that was going to be. And I don’t think patients could possibly have realised that when they agreed to that happening at the beginning, do you know what I mean?” (Ed D2)

“I know that when people signed up for the trial they knew that they might not stay on the pump. But I suspect that people at that point wouldn’t really have understood just what an impact that pump has on their life. So I can understand if somebody was told they couldn’t keep their pump that they’d be very upset or angry about it.” (Ed F4)

As well as worrying that patients would be upset or angry, some staff expressed a strong sense of personal discomfort about the prospect or possibility of withdrawing treatment from patients who liked it and felt they were benefitting from it and who, as Ed E1 further noted, had also given their time to take part in the trial:

“I can see it’s maybe quite cruel to give somebody a treatment which they feel makes their life much easier, and then try and take it away from them.” (Ed D2)

“It was kind of frustrating because they were helping in a study and you were giving them something which they felt was a benefit to them and then you were going to take it away as soon as the study stopped. So it wasn’t, you know, I don’t know, it just didn’t feel right to be doing that.” (Ed E1)

### Experiences of withdrawing the pump

In line with these staff members’ expectations and concerns, patients from whom pump therapy was withdrawn were described as having been “disappointed” (Ed G), “upset” (Ed A2), “disgruntled” (Ed A1) and “gutted” (Ed G) with staff, including Ed G, also noting the personal upset and distress they had themselves experienced as consequence:

“Certainly, he was gutted, you know, I basically just didn’t give it back to him. And I gave him the prescription for insulin inject- pens and discussed what to start on and how to go- and he was like looking at me like a, you know, a rabbit caught in the headlights. And I’m thinking: ‘oh god, this is really awful.’” (Ed G)

However, some staff also reflected upon how, while often upset and disappointed, most patients had also been stoical and accepting of the decision, especially when these staff members (*n* = 5) had been involved in a 6-month pilot study that had preceded the main trial. As these staff noted, their earlier exposure to individuals who had been angry and distressed about the removal of pump therapy at the end of the pilot study had alerted them to the need to devise proactive strategies in the main trial to prevent or manage patients’ (potential) upset and anger at its end:

“We’d learnt a lesson from, I think the pilot of having a situation where people were really upset about not being able to keep it despite signing things, having it written. It was very difficult having somebody in front of you, crying and getting frustrated and saying all sorts of things to you, so we were much clearer we think this time round with, just reminding people of the rules and we tried to do it each time we met them as well, reminding them this was a trial and if they don’t meet NICE criteria [at the end] the pump would go back.” (Ed F3)

While some staff, including Ed F3, highlighted the benefits of reminding patients throughout the trial that pump therapy could be withdrawn so that their expectations were managed from the outset, others (from site A), described how they had offered patients a ‘wash out’ period at the end of the trial to help them adapt psychologically and practically to removal of the pump:

“We knew we were going to have difficult conversations, so we came up with plans of how we could best do it, so we didn’t just switch them there and then on the 24 month visit. We said- we reminded them—we had kind of—you know a few people were quite upset and grumpy about it. And we said: ‘look we have some kit we can give you that you can—we can tide you over for another month or six weeks, while we sort out your pens and getting you back—to switch you back onto MDI and doing it in a supportive as way as possible’. We didn’t, you know, rip the pump off them at that appointment and say: ‘there’s your pens back, off you go’. And so we just—having that discussion at the meetings before for all of them just helped us come up with a kind of individual plan to sort of damage limitation really.” (Ed A1)

While such strategies were described as “damage limitation”, staff also noted that they had been relatively effective in so far as, “although we had some people who are a bit upset, I don’t think anyone was so disgruntled that he had to go and fetch [diabetes consultant’s name] to come and smooth things over” (Ed A1). Indeed, in cases where these strategies were not employed, due to lack of advance planning and poor communication within and across teams, staff described encountering much more extreme emotional reactions from some patients. While these staff, like their colleagues, had anticipated that patients would be upset, they described themselves as having been “utterly ill-prepared” (Dr F) for the anger and distress that they actually encountered. This included Dr D who described having felt “overwhelmed” during a consultation where they had had to inform a patient that pump therapy would be withdrawn and that Dr D described as having been “one of the most uncomfortable things I’ve done for a long time”. As Dr D went on to elaborate:

“I don’t often have negative interactions with people, but he was very cross and very angry with me, and understandably I guess, maybe, you know, he felt passionately about what he was using. But it was uncomfortable and I don’t normally feel uncomfortable in a consultation…But, on this occasion, I felt really sort of overwhelmed by his strength of feeling.” (Dr D)

A similar incident was reported by Dr G1, who described having been taken aback by the degree of distress and anger exhibited by one individual from whom they had withdrawn pump therapy:

“The one locally that really didn’t go well, was a gentleman whose control had got worse on the pump. He’d had two admissions with DKA [diabetic ketoacidosis —a life threatening condition]. And I was explaining that in fact on balance it was actually more dangerous for him to remain on a pump, cause he was more likely to be admitted in DKA again on a pump, because he’d not had any DKAs off the pump. And he was the one that walked out. He didn’t shout or give me any indication. He just stood up and said: ‘okay’ and walked out. So obviously he was boiling underneath and I hadn’t quite appreciated this. And then his wife just looked at me cause she was totally shocked when he walked out as well.” (Dr G1)

In site F, where, due to limited communication within the team, only some patients had been ‘primed’ for closeout, Dr F also described very negative experiences of withdrawing treatment, albeit in this site, the full extent of patients’ anger did not become fully apparent until after the end of the trial appointments had taken place:

“And I felt that the conversations went reasonably well, because it was a rational discussion between adults. I pointed out why I was not recommending continuation. And I also said very explicitly: ‘you know, if this doesn’t work, come straight back’. And then I started to get these messages from my colleagues that they were being bombarded with emails about how this was terrible. And that’s when it all—that’s when, that’s when we realised there was a problem.” (Dr F)

Elsewhere in their interview, this health professional talked about how their colleagues had never actually given them sight of the emails or separate a letter of complaint received from another patient, “possibly because they were being protective of me, or because the patients had said they didn’t want them to show them me, but I know they got a lot of abuse from these patients.” (Dr F).

As Dr F, Dr G1 and Dr D’s accounts make apparent, not only did some patients exhibit reactions that they had found very upsetting, they also interpreted patients’ anger and/or subsequent complaints as signalling strongly that that these patients had felt that they had failed them in their duty of care. Understandably, to try to make sense of these kinds of experiences, this group of individuals tended to provide much more reflective accounts than colleagues whose end-of-trial encounters had been more straightforward. In doing so, these individuals not only speculated that patients had gained unanticipated quality of life benefits from using a pump, but they also reflected upon the possibility that some might have held a misconception that the (pump) treatment to which they had randomised had been needs rather than hypothesis driven—a phenomenon termed the ‘therapeutic misconception’ in the literature [21]. In developing this line of thinking, Dr G1, for instance, drew a potential parallel between perceptions and views of REPOSE participants and those of patients who had taken part and a recently completed trial (the 5x1 study). In the latter, as Dr G1 noted, embedded psychosocial research [22, 23] had shown that many participants had held the view that the trial arm to which they had been randomised had enabled them to access the treatment of greatest personal and clinical benefit:

“We didn’t actually do quite the same thing as we did with 5x1 where we asked them which arm [DAFNE delivered over one week, or DAFNE delivered one day a week over five weeks] they thought was best…In the 5x1 88% said: well this [the arm to which the participant was randomized] was the best way I can’t imagine it in the opposite way. And I think if we’d asked that question here we might have got a similar result because the people just rationalised that whatever arm they were randomised to that was how it was meant to be.” (Dr G1)

Hence, as Dr G1 indicated, not only could a (mis)conception that treatment allocation had been needs driven potentially help explain patients’ anger when they had attempted to withdraw it, but also the difficulties staff had encountered convincing some individuals that they had not gained clinical benefit from using a pump. A similar conclusion was reached Dr F, who also drew upon learning from the 5x1 trial to suggest that a therapeutic misconception might help explain why some patients appeared to have struggled to separate the clinical benefits of using an insulin pump from the clinical benefits gained from having attended the structured education (DAFNE) course at the start of a trial:

“…the study that looked at DAFNE given over five days or given over five weeks × the patients at the end of that were absolutely adamant that the way they did it was the best way to do it. And I guess the pump [REPOSE trial] is the same. So we, because DAFNE is good, you think that your [pump or MDI] DAFNE is the way to do it. And I guess that’s what this was. I mean you know, DAFNE, it was fantastic I loved it. What I loved was DAFNE with pump. And as a patient I didn’t distinguish between DAFNE and pump, they’ll have attributed their benefit to the pump, whereas the MDI group will have attributed their benefit to DAFNE. Now that’s only just occurred to me in talking to you. So, what I thought I was doing was having an intellectual conversation saying: ‘well actually if there is no clear indication for a pump, they should try without it’ but that’s not what their perception will have been; to the person with diabetes there would have been a feeling of, you, know what I now regard as my lifeline is being taken away from me.” (Dr F)

### Side stepping ethical issues and emotional challenges at the end of the trial

Given their concerns about upsetting and angering patients, it is perhaps unsurprising that staff who belonged to sites where there was easy access to pump funding at the time of closeout did not generally withdraw pump therapy unless there was a compelling health or safety reason to do so. Indeed, staff in these sites noted that, by keeping most/all patients on pumps, they had been able to side step the anguish and upset (both to patients and themselves) that colleagues in other sites had had to confront:

“It would have been—I’ll be honest—I would have been devastated for them…I think if you’ve done really well with something and you know, you’ve worked with the team, and worked really hard and then you’ve had that [pump] taken away from you, I think that would be awful. And I think, as an educator, I’d have found that awful as well.” (Ed D2)

“Well I guess if we had been in the same situation as other centres where there was no funding stream to continue patients, we would have had to say, ‘sorry, we don’t have any money for you to continue on this.’ And I think it would be very difficult. I mean obviously I would imagine in other places it’s caused a bit of damage to the doctor or healthcare professional relationship because there was a sort of building up of trust going through the study and at of the end of it.” (Dr B)

Indeed, because of their concerns about damaging on-going therapeutic relationships, such staff, including Dr B, were keen to emphasise that, had they removed pumps from individuals when it was possible to access on-going funding, they would have failed them in their duty of care. However, even in those sites where it had been possible to keep patients on pump therapy, staff still felt they had not been able to bypass or address ethical issues altogether. Specifically, staff shared their frustrations and worries that, because the trial protocol had not permitted them to tell patients that they could remain on pump therapy until after final trial data had been collected, patients had experienced what they saw as avoidable worry and distress:

“Well I think definitely they should have known earlier that they were continuing on the pump. Whether that was at the beginning, or halfway through, but definitely not leave them for two years thinking you know, ‘I’m not going to have this pump at the end of this.’ So I think that was a bit cruel, cos it might have given them peace of mind.” (Ed E1)

Indeed, as some staff further noted, the requirement to follow the protocol had also presented emotional challenges, requiring them to undertake emotion work to try to keep patients calm:

“…they were very worried I think most of the patients who came in. We kind of almost had to calm folk down a little bit. We had quite a few were walking in the door at the final visit very, very scared because they knew it was the end of the trial and they didn’t know what was going to happen…I was really trying to bite my lip actually and not say to people you’ll be alright, but I was not saying that, and it was hard, trying to be good and do the trial properly.” (Ed C2)

### Lessons learned from REPOSE: improving closeout of future trials

“I suppose we should have discussed it more because this trial has made us think much more about what happens at the end and I actually think for ethical reasons perhaps we should have discussed it more because just because they’ve finished the trial it doesn’t mean there aren’t ethics around it…and thinking about it, one thing we should have thought about is, you know, preventing patients from getting cross.” (Dr G2)

When they reflected back on their experiences, most staff expressed the view that the trial could have been ended in a more carefully planned and ethically robust way. This, as various staff members pointed out, was not only to avoid undue distress being caused to patients (and arguably also to themselves), but also, as some individuals noted, to promote great consistency in practices across trial sites and, hence, alleviate their concerns that patients had not received equal treatment at the end of the trial.

To improve end-of-trial conduct and develop more proactive and consistent approaches, staff highlighted the importance of having earlier discussions involving representatives from all of the sites. Indeed, some noted that, had such discussions taken place, they might have considered employing the ‘priming’ and ‘wash out’ strategies developed by those who had been involved in the pilot:

“What I would do differently, I think we should have shared views, I think a discussion along this line at a trial management groups, we might really have begun to talk about what the scenarios might be and shared views.” (Dr B)

Staff also suggested that discussions should continue after closeout had begun (e.g. through regular teleconferences) to enable examples of good practice to be identified, develop vignettes with could be used for role-play and training of future staff, and learn from situations where conduct might be improved. With regards to the latter, staff were keen to share their experiences of, and learning from, REPOSE, particularly those who felt, with hindsight, that end-of-trial appointments had been mismanaged within their sites. Specifically, some staff suggested that a lack of communication and planning within their teams—and also the lack of clear and detailed guidance in the SOP—had resulted in these appointments having been conducted by individuals (mostly lead clinicians) who had little or no knowledge of the patients concerned. As a consequence, as Dr F reflected, such individuals had not been cognizant of the issues that had come up for patients during the trial and, hence, had not always managed closeout appointments in careful and sensitive ways:

“I just assumed everyone had come because they wanted to do the research and isn’t that nice I get to play with it—see that’s how I thought of it—the advantage is I might get to use a pump for two years and see what it’s like. And that’s how I thought of it when I went into the appointments, and these patients didn’t think of it like that at all.” (Dr F)

As some staff, including Ed F2, further suggested, receiving the news that pump therapy was to be withdrawn from someone who was ostensibly a stranger, and who did not necessarily have a comprehensive knowledge of one’s clinical history and personal circumstances, might also have heightened patients’ negative experiences at the end of the trial:

“…when they came back to see the doctor at that point, that may for some of them have been the first time they’d met that doctor as well. So it may have been difficult for them in that respect to be told at that point that ‘actually we don’t feel that the pump is necessary for you’—I think some of them found that quite hard to hear as well, the fact that they’re meeting someone for the first time who didn’t actually know them and they’re saying ‘actually you don’t meet the criteria.’” (Ed F2)

Hence, staff recommended use of a team-based approach in future trials, with several individuals, including those who patients had got to know well during the trial (and hence who patients trusted), being involved in the end-of-trial appointments. Such an approach, as Dr G intimated, might help to prevent an on-going therapeutic relationship from being compromised:

“I would like to think that whoever is terminating the study had been involved throughout, I think that would make all the difference…And the last session should have been a co-ordinated session in which they were with their usual [diabetes health professional] —cause even then if I’d come in as ‘this is the doctor’, they’d have been with an educator who knew them, and it would have been a very different experience for them, it would have meant more continuity of clinical support. We should have turned the visit into, ‘there is a team to support you’ and that wasn’t there.’” (Dr F)

Some staff also suggested that a team-based approach could present opportunities for staff who were anxious about withdrawing treatment to receive support from colleagues. In addition, as Dr G2 noted, when colleagues had been present in the room who had known patients during the trial, this had proved helpful because they had been able to give advance warning of those who were likely to be upset and angry about withdrawal of treatment:

“I think, she [educator] knew some of the patients better than I did because she’d done the course with some of them. And so she was warning which ones might be tricky. And you know, she is a good judge of character. So that was really helpful.” (Dr G2)

Staff also noted that, to achieve an integrated, team-based approach, funding for dedicated time should be built into future trials because, as Ed F2 explained:

“I think one of the issues was as well is, because we’re such busy clinical team and this was kind of fitted in as part of our clinical work. So we didn’t have time to do things properly.” (Ed F2)

To promote more consistent, proactive practices, staff also suggested that, in the future, they would value a high level of external co-ordination and support (e.g. from a CTU) and a more detailed SOP outlining closeout procedures—one that drew upon learning from the REPOSE trial:

“I think everyone should have been advised to have those difficult conversations, you know, about not being able to keep the pump at the end, being advised to do that at every meeting and every opportunity so people had their expectations managed…Maybe a reminder to meet and discuss who was going to stay on pumps, and who wasn’t, you know, just a reminder to say, ‘have you had that conversation within your team? Has your patient been primed?” (Ed F3)

Alongside practical and planning issues, and given their concerns that distress had been caused to some patients, staff were also keen to suggest that, in future trials, closeout procedures should be subjected to much higher levels of ethical scrutiny. Indeed, as Dr G1 noted, while the recruitment phase of trial is subjected to careful ethical oversight to help safeguard the rights and wellbeing of patients, a similar level of thought, planning and accountability is not currently required at the end of a trial:

“When you’re writing a protocol, the major thing is: can we get enough people into the study? That’s always the major hurdle isn’t it…with getting ethical approval. And, in hindsight, we probably ought to have sorted the close-out in more detail, and put time aside to do this with amendments (to the ethics committee) or whatever it needed.” (Dr G1)

Indeed, it was noted by this individual as well as several others that, when the trial protocol had been reviewed by the ethics committee, no questions and concerns had been raised by the potential withdrawal of treatment from patients at the end of the trial and how this would be managed.

## Discussion

This study sought to address a neglected area in the trials literature: staff experiences of closing out a clinical trial that entailed withdrawal of treatment—insulin pumps—from some patients. In doing so, we have shown that, like the recruitment and implementation phase [2–14], the ending of a trial may bring myriad practical, ethical and emotional challenges to the fore for staff. We have also shown that, in the absence of clear guidelines and advance planning, staff attempted to address these challenges in a variety of different ways, some of which were seen to intensify their ethical concerns, principally those about whether patients had been given fair and equal treatment at the end of the trial.

At the trial’s planning stage, and when the SOP for closeout was circulated, it was anticipated that pump therapy would be withdrawn at the end, unless patients met clinical criteria required for NHS funding. In actuality, however, staff rarely withdrew treatment unless a patient wanted this to happen, except in those sites where access to local NHS funding was very restricted and clinical criteria had to be strictly adhered to. Whether or not they decided to withdraw pump therapy, staff appeared to find themselves caught in a similar ethical and moral maze to that observed by Zussman in a study of how triaging decisions are made in intensive care units in the USA where demand for beds outstrips supply [24]. Specifically, staff found themselves having to balance the interests of individuals who, by virtue of taking part in REPOSE, had become attached to a therapy that they then wanted to retain against those of a wider clinical (i.e. non-trial) population, which included individuals who more readily met clinical criteria for funding. Zussman has presented this conflict as being one between Hippocratic individualism and utilitarianism—a conflict that, he suggests, threatens the very nature of the doctor-patient relationship [24]. In the trial context, this conflict could equally be understood as one between doing research and delivering clinical care, not least because research and clinical care are themselves driven by the conflicting paradigms of utilitarianism and Hippocratic individualism. While there was some disagreement between staff about whether an ethic of utilitarianism or Hippocratic individualism should guide decision-making at the end of the trial, most saw their duty of care to REPOSE participants as having been paramount. Indeed, staff who decided to keep most/all patients in their sites on pumps emphasised that a desire to preserve on-going therapeutic relationships had been central to their decision-making, whereas those who had withdrawn pump therapy noted damage to the therapeutic relationship as a consequence and highlighted the importance of this relationship being better protected in future trials involving withdrawal of treatment. Whatever the approach taken at the end of the trial, staff felt that the closeout of future trials could be conducted in more ethically robust and considered ways. To achieve this, most also suggested this aspect of trial work should be subjected to greater ethical scrutiny and oversight.

While clinical trials are subjected to external ethical oversight, the recruitment phase, as Petryna [17] has observed, tends to be where most attention, reflection and ethical debate are focussed, this also being a concern voiced by Fisher in a powerful and thought-provoking exposition of the pharmaceutical trials industry in the USA. In this, she notes that bioethical reflection tends to concentrate on helping to ensure consent to take part in a trial is given freely and willingly to the possible detriment of what happens later on in the trial: “Oftentimes, bioethicists are concerned about reducing the coercion of prospective human subjects, but there is also a danger in exploiting individuals if they are not sufficiently rewarded for their investment of time and exposure to risk” (p 125) [10]. In keeping with these kinds of concerns, staff in our study shared their worries that upset and distress had been caused to some individuals at the end of the trial, especially those from whom treatment was withdrawn. Most staff also expressed the view that, had there been more advanced planning and discussion, and greater accountability to an ethics committee, some of this distress could have been reduced, if not avoided altogether. Hence, staff saw their participation in this interview study as an important opportunity to raise awareness of the ethical and other difficulties they had encountered and expressed the hope that the findings would be used to help improve the (ethical) conduct of future clinical trials involving withdrawal of treatment.

A key finding from this study is that some of the same ethical issues that may vex staff at the outset of the trial may carry over to, or re-present themselves at, its end as well as there being potential for new ethical considerations and challenges to arise. For instance, when staff tried to make sense of patients’ upset, anger and distress, they reflected upon the possibility that some individuals who were most resistant to withdrawal of pump therapy might have been under the influence of the therapeutic misconception [21], a phenomenon that has been observed in empirical research undertaken with patients [25, 26]. As staff observed, this was because REPOSE participants appeared to hold strong and recalcitrant views that the (pump) treatment to which they had been allocated had been most therapeutically suited to them. Indeed, it was noted that, if patients did believe that they had received treatment that had been needs driven, it was entirely understandable that they might have felt staff had failed them in a duty of care by withdrawing this treatment at the end of the trial. While other patients, as staff suggested, might not have initially seen pump therapy as being a better treatment than an injection regimen, many appeared to shift their perceptions as the trial progressed by virtue of having experienced unanticipated (non-clinical) benefits from using a pump. This issue, as staff pointed out, had had important, and initially unanticipated, ethical and emotional repercussions. First, because it led them to question whether patients’ consent to take part in a trial involving possible withdrawal of treatment remained valid at the end, a question that has prompted others to recommend researching patients’ ‘whole-trial’ experiences to better understand the unanticipated (ethical) harms, as well as benefits, that may arise from trial participation [26]. Second, because some of the patients whom staff had closed out from the trial expressed the view that the non-therapeutic benefits they had gained from using a pump should guide treatment decision-making, rather than this being driven by formal clinical criteria. Indeed, although only caveated interpretations can be drawn from staff’s accounts, it is possible that patients might have been experiencing a form of therapeutic misconception at the end of the trial in so far as they appeared to expect staff to tailor treatment to their personal needs rather than to follow a standardised protocol. It is regrettable that it was not possible to interview patients for this study because of the lengthy time required to obtain the necessary approvals. However, research undertaken by Wynne [27] with people with multiple sclerosis who took part in a trial of hyperbaric oxygen therapy appears to support staff’s speculations and concerns. Wynne observed that most patients wanted to remain on this therapy, despite the trial failing to show clinical efficacy, because they saw themselves as having gained benefits that were substantively different from those that the trial set out to formally assess. Notably, in Wynne’s study, patients felt so strongly that they should remain on hyperbaric oxygen therapy that they made independent financial arrangements for treatment following the trial.

Conflicts between research and clinical care, which have been shown to vex and challenge staff at earlier stages in the trial process [5, 8], with ensuing emotional consequences [7, 16], also appeared to re-present themselves at the end of the REPOSE trial. For instance, some staff expressed their concerns that they had failed patients in their duty of care because the trial protocol had not permitted them to disclose treatment decisions until after final trial data had been collected. Others voiced frustrations that, because of the requirement to follow the trial protocol, they had been unable to offer patients optimal and individualised clinical care, which might have enabled them to gain greater clinical benefit from using a pump. To address this concern, staff also described allowing some patients to remain on pump therapy following the trial, despite these individuals having not met clinical eligibility criteria. Indeed, a desire to reconcile the conflict between research and clinical care appeared paramount to staff decision-making in these specific instances, and more generally when patients were allowed to keep their pump, with staff emphasising that they would have failed patients in a duty of care had they removed pumps when it was possible to access on-going funding. While these staff thus presented powerful and convincing ethical justifications for their decision-making, which principally cohered around their desire to preserve on-going therapeutic relationships, it is also possible that they might have used these justifications as post-hoc rationalisations to present themselves in a morally favourable light [28]. Rather than speculating further on this potentially delicate matter, what is perhaps more helpful and salient to note is that the (ethical) conduct of these staff was questioned by other members of the co-investigator team; especially those who had followed the protocol and withdrawn treatment from patients who did not meet clinical eligibility criteria. These individuals speculated that, as well as prioritising the interests of individual patients over those of the wider diabetes population (an ethical standpoint that was sometimes antithetical to their own), their colleagues might have been attempting to avoid the physical and emotional work involved in reverting patients back to injection regimens. In doing so, these individuals not only raised important questions about whether patients across the sites were treated fairly and equally at the end of the trial, their accounts also made apparent that inconsistent practices could be a source of friction within the trial team.

## Conclusion

The Declaration of Helsinki now requires that “researchers and host country governments should make provisions for post-trial access for all participants who still need an intervention identified as beneficial in the trial” [29]. While this is clearly an important step towards safeguarding patients’ rights and interests, important questions remain about those individuals who do not gain clinical benefit from a trial intervention but who nonetheless feel they have gained personal benefits. As the findings of this study powerfully highlight, there may be no simple answers or easy solutions to offer—indeed, staff found themselves torn between an ethic of Hippocratic individualism and one of utilitarianism. Clearly only limited conclusions can be drawn from one study, and it is vital that further research be undertaken on staff and patients’ closeout experiences. However, a key learning point from the REPOSE trial is that more thought, planning and ethical oversight should go into the design and conduct of future trials involving withdrawal of treatment(s); indeed, staff were keen to cascade their experiences with this agenda in mind. In particular, staff wanted others to learn from examples of what had worked in their particular sites (e.g. priming patients for withdrawal of treatment; offering wash out strategies to allow patients to adjust practically and psychologically to treatment withdrawal) as well as those that had not (e.g. having an individual rather than a team involved in the appointments where treatment was withdrawn). While, in doing so, staff emphasised their ethical responsibilities to their own and future patients, we would argue that there should also be an ethical responsibility to trial staff to offer them better guidance, input and support. Indeed, as our interviews made only too apparent, not only did the ending of the trial cause distress to some patients, it also had a significant emotional impact on staff, leaving some feeling isolated, overwhelmed, unsupported and sometimes also resentful.

## Additional file

Additional file 1: Table S1. Participant characteristics. (PDF 341 kb)

## Abbreviations

CCG: Clinical Commissioning Group
CTU: Clinical Trials Unit
DAFNE: Dose Adjustment for Normal Eating
DKA: Diabetic ketoacidosis
MDI: Multiple daily injections
NHS: National Health Service
NICE: National Institute of Clinical Excellence
RCT: Randomised controlled trial
REPOSE: Relative Effectiveness of Pumps Over MDI and Structured Education
SIGN: Scottish Intercollegiate Guidelines Network
SOP: Standard operating Procedure
