# Supplementary material for: Staff experiences of closing out a clinical trial involving withdrawal of treatment: qualitative study
Source: Trials. 2017 Feb 7;18:61. doi: 10.1186/s13063-017-1813-y (PMC5297163; doi:10.1186/s13063-017-1813-y)
Supplement: Additional file 1: Table S1. — Participant characteristics. (PDF 341 kb) [file 13063_2017_1813_MOESM1_ESM.pdf]

Table 1: Participant characteristics. For Diabetes Consultants and Educators and their experience of providing pump therapy.

	**N**	***%***
REPOSE SITES (n=7)		
Interviewees per site (range)	1-5	
Interviewees per site (mode)	3	
ROLE		
Diabetes Consultants	7	33
Educators	14	66
PUMP THERAPY EXPERIENCE		
< 5 years	5	24
5-10 years	9	42
>10 - 15 years	7	34
